# Overeating Saturated Fat Promotes Fatty Liver and Ceramides Compared With Polyunsaturated Fat: A Randomized Trial

**DOI:** 10.1210/jc.2019-00160

**Published:** 2019-08-01

**Authors:** Fredrik Rosqvist, Joel Kullberg, Marcus Ståhlman, Jonathan Cedernaes, Kerstin Heurling, Hans-Erik Johansson, David Iggman, Helena Wilking, Anders Larsson, Olof Eriksson, Lars Johansson, Sara Straniero, Mats Rudling, Gunnar Antoni, Mark Lubberink, Marju Orho-Melander, Jan Borén, Håkan Ahlström, Ulf Risérus

**Affiliations:** 1 Department of Public Health and Caring Sciences, Clinical Nutrition and Metabolism, Uppsala University, Uppsala, Sweden; 2 Department of Surgical Sciences, Radiology, Uppsala University, Uppsala, Sweden; 3 Antaros Medical AB, BioVenture Hub, Mölndal, Sweden; 4 Department of Molecular and Clinical Medicine, Institute of Medicine, Sahlgrenska Academy at University of Gothenburg, Gothenburg, Sweden; 5 Division of Endocrinology, Metabolism and Molecular Medicine, Northwestern University, Chicago, Illinois; 6 Department of Medical Sciences, Uppsala University, Uppsala, Sweden; 7 Wallenberg Centre for Molecular and Translational Medicine and Department of Psychiatry and Neurochemistry, University of Gothenburg, Gothenburg, Sweden; 8 Department of Public Health and Caring Sciences, Geriatrics, Uppsala University, Uppsala, Sweden; 9 Center for Clinical Research Dalarna, Falun, Sweden; 10 Science for Life Laboratory, Department of Medicinal Chemistry, Uppsala University, Uppsala, Sweden; 11 Metabolism Unit, Endocrinology, Metabolism and Diabetes, and Integrated CardioMetabolic Center, Department of Medicine, Karolinska Institutet at Karolinska University Hospital, Huddinge, Stockholm, Sweden; 12 Department of Medicinal Chemistry, Uppsala University, Uppsala, Sweden; 13 Department of Clinical Sciences in Malmö, Lund University Diabetes Centre, Lund University, Malmö, Sweden

## Abstract

**Context:**

Saturated fatty acid (SFA) vs polyunsaturated fatty acid (PUFA) may promote nonalcoholic fatty liver disease by yet unclear mechanisms.

**Objective:**

To investigate if overeating SFA- and PUFA-enriched diets lead to differential liver fat accumulation in overweight and obese humans.

**Design:**

Double-blind randomized trial (LIPOGAIN-2). Overfeeding SFA vs PUFA for 8 weeks, followed by 4 weeks of caloric restriction.

**Setting:**

General community.

**Participants:**

Men and women who are overweight or have obesity (n = 61).

**Intervention:**

Muffins, high in either palm (SFA) or sunflower oil (PUFA), were added to the habitual diet.

**Main Outcome Measures:**

Lean tissue mass (not reported here). Secondary and exploratory outcomes included liver and ectopic fat depots.

**Results:**

By design, body weight gain was similar in SFA (2.31 ± 1.38 kg) and PUFA (2.01 ± 1.90 kg) groups, *P* = 0.50. SFA markedly induced liver fat content (50% relative increase) along with liver enzymes and atherogenic serum lipids. In contrast, despite similar weight gain, PUFA did not increase liver fat or liver enzymes or cause any adverse effects on blood lipids. SFA had no differential effect on the accumulation of visceral fat, pancreas fat, or total body fat compared with PUFA. SFA consistently increased, whereas PUFA reduced circulating ceramides, changes that were moderately associated with liver fat changes and proposed markers of hepatic lipogenesis. The adverse metabolic effects of SFA were reversed by calorie restriction.

**Conclusions:**

SFA markedly induces liver fat and serum ceramides, whereas dietary PUFA prevents liver fat accumulation and reduces ceramides and hyperlipidemia during excess energy intake and weight gain in overweight individuals.

## Teaser

We show the importance of dietary fat type on liver fat accumulation and ceramides during overfeeding-induced weight gain in 61 overweight men and women.

Ectopic fat, especially liver fat accumulation and nonalcoholic fatty liver disease (NAFLD) are directly associated with multiple metabolic disturbances, *e.g.*, dyslipidemia and insulin resistance (1). NAFLD is partly determined by genotype, but lifestyle is key to prevention and treatment. Although excessive energy intake is a key driver of NAFLD, surprisingly, few studies have been conducted in humans on the role of dietary composition in ectopic fat accumulation. Dietary fatty acids can modulate fat distribution, independent of changes in body weight, as we previously showed that a diet rich in omega-6 polyunsaturated fatty acids (PUFAs) compared with saturated fatty acids (SFAs) reduced liver fat content in abdominally obese subjects during isocaloric conditions (2). Furthermore, we recently showed that PUFA compared with SFA also could prevent accumulation of liver fat and visceral adipose tissue (VAT) in young, lean subjects during overfeeding (3). Potential effects of dietary fat type on other ectopic fat depots of potential clinical relevance, such as pancreas fat, are unknown. Whether the increase in liver fat content in response to dietary SFA is a result of an increase in hepatic palmitate uptake or endogenous hepatic metabolism [such as *de novo* lipogenesis (DNL)] is unknown, but results from animal studies generally show suppressive effects on DNL by PUFA vs SFA (4–6). Furthermore, fatty acid desaturation by the enzyme stearoyl-coenzyme A desaturase (SCD) may be important for liver fat accumulation, as indicated in experimental studies (7, 8), and the SCD activity index increased by dietary SFA (vs PUFA) in intervention studies (2, 3).

Bile acids (BAs) have broad effects on hepatic triglyceride and cholesterol metabolism and have been suggested to play a central role in the development of NAFLD (9). The cholesterolemic effect of SFA is well established, but whether SFA also exerts detrimental effects on BA metabolism and if this plays a role during SFA-induced liver fat accumulation are unknown.

Luukkonen and colleagues (10) recently suggested that SFA-induced liver fat accumulation in humans may be a result of increased lipolysis, potentially mediated via inflammation-related pathways in adipose tissue. In keeping with such data, a recent study in rats showed lower inflammation and oxidative stress after a diet rich in PUFA vs SFA (11). However, concern has been raised that a high intake of *n*-6 PUFA also increases inflammation, although this is based mainly on experimental studies and theoretical assumptions with little supporting data in humans (12). Animal studies suggest a potential causal role of ceramides, a group of sphingolipids, in diet-induced NAFLD (13, 14) and that a reduction in ceramides leads to reduced hepatic steatosis and lipogenesis (14, 15). Dietary fat-specific effects on circulating ceramides have recently been suggested in humans (10).

Here, overweight individuals were overfed with either SFA (palm oil) or omega-6 PUFA (sunflower oil) with the hypothesis that PUFA would counteract the accumulation of liver fat during 2 months of controlled weight gain and that SFA-induced liver fat accumulation is a result of increased hepatic uptake of palmitate. We further aimed to investigate whether effects on liver fat accumulation may be related to changes in sphingolipids (*e.g.*, ceramides in serum and adipose tissue).

## Methods

### Participants

Overweight men and women were recruited by local advertising. Inclusion criteria included participants ages 20 to 55 years and body mass index (BMI) of 25 to 32 kg/m^2^; exclusion criteria were the following: diabetes (fasting glucose >7 mM at two occasions) or liver disease; pregnancy; lactation; alcohol abuse; claustrophobia; abnormal clinical chemistry test results; use of drugs influencing energy metabolism; use of omega-3 supplements or extreme diets; regular heavy exercise (>3 h/wk); intolerance to gluten, egg, or milk protein; and implanted metals. Subjects were instructed to maintain their habitual diet and physical activity level throughout the study. Subjects were fasted overnight (10 to 12 hours) and were discouraged from physical exercise and alcohol intake 48 hours before measurements.

### Study design

The LIPOGAIN-2 study was a 12-week, double-blind, parallel-group, randomized trial in free-living subjects, carried out from August 2014 through June 2015 at the Uppsala University Hospital, Uppsala, Sweden. Subjects were randomized by computer-generated lists (generated by a statistician not otherwise involved in the project), stratified by sex, age, and BMI. Double-blinding was ensured by labeling, and the code was concealed from all investigators until study completion and statistical analyses of primary outcomes. During the first 8 weeks, subjects were overfed (weight gain), followed by 4 weeks of caloric restriction (weight loss). The trial was registered at www.clinicaltrials.gov as NCT02211612.

### Dietary intervention

Sixty-one participants were randomized to eat muffins containing either sunflower oil (high in PUFA, linoleate, 18:2n-6) or palm oil (high in SFA, mainly palmitate 16:0) during 8 weeks. Both oils were refined; fatty acid composition has been described (3). Body weight was measured, and muffins were provided to subjects weekly at the clinic. Muffins were baked in large batches under standardized conditions in a metabolic kitchen at Uppsala University. Muffins were added to the habitual diet (to be consumed anytime during the day) and individually adjusted weekly (altered by ±1 muffin/d depending on rate of weight gain) to achieve a 3% weight gain (on average, 2.9 ± 0.5 muffins were added, equaling ∼40 g of oil/d). Except for fat type, muffins were identical in composition (51 energy% fat, 44 energy% carbohydrates, and 5 energy% protein). Following the overfeeding period, subjects switched to a 4-week, low-calorie diet, consisting of ∼800 kcal/d (∼52 energy% carbohydrate, ∼26 energy% protein, and ∼18 energy% fat; Modifast; Nutrition & Santé).

### Fat depots and body composition

Liver and pancreas fat content, liver volume, and volume of total body and visceral fat were assessed by MRI using a 1.5T Achieva clinical scanner (Philips Healthcare), modified to allow arbitrary table speed. Collection and analyses of the MRI data were performed at one center under blinded conditions. A single operator, trained by an experienced radiologist, performed all measurements. The absolute amount of liver fat in liters was calculated by the multiplication of liver volume by liver fat content (%). Total body and visceral fat were quantified using a previously described automated image analysis algorithm (16). Total body fat was also measured using whole-body air displacement plethysmography (ADP; Bod Pod; COSMED®), according to the manufacturer’s instructions. Total body water content was measured by bioelectrical impedance analysis (Tanita BC-558; Tanita Corporation). Data from the ADP were corrected for total body water content by using a three-compartment model (17).

### Palmitate uptake

In a subgroup (n = 5 in each group), palmitate uptake in liver, pancreas, heart, and skeletal muscle was measured on a 3T positron emission tomography (PET)-magnetic resonance (MR) scanner (Signa PET/MR; GE Healthcare). A single bed-position dynamic PET scan, covering the splanchnic bed, was performed during 60 minutes, starting simultaneously with a bolus injection of [^11^C]palmitate (361.35 ± 11.9 MBq). Regions of interest for liver, pancreas, and skeletal muscle tissue, as well as the blood pool in ascending aorta and portal vein, were outlined manually based on the anatomical information in a T1-weighted MR image acquired simultaneously with the PET scan. Myocardium and left-ventricular cavity were segmented automatically on PET images using the cardiac module in Carimas. Quantification of [^11^C]palmitate uptake in liver, pancreas, and skeletal muscle was performed using an image-derived input function based on average radioactivity concentrations in an ascending aorta and portal vein, corrected for radioactive metabolites, using the measured parent fraction remaining in plasma at 10, 20, 30, 40, and 50 minutes postinjection, as well as plasma to whole-blood radioactivity concentration at 5, 10, 20, 30, 40, and 50 minutes postinjection. Four different parameters were calculated based on the [^11^C]palmitate data:

1. The accumulation rate constant [influx constant (K_i_)] was estimated using Patlak analysis for the 5- to 60-minute postinjection interval.
2. Standardized uptake values (SUVs) were estimated, adjusting radioactivity concentrations for intersubject differences in body weight and administered amount of [^11^C]palmitate [SUV = radioactivity concentration (becquerel/mL)/body weight (gram) × administered tracer (becquerel)] for the uptake windows of 30 to 35 minutes and 55 to 60 minutes postinjection.
3. Myocardial fatty acid uptake was assessed by the multiplication of the plasma nonesterified fatty acid (NEFA) concentration by the fractional uptake rate (determined by the division of the myocardial concentration peak value by the integral of the metabolite-corrected plasma curve).
4. Myocardial fatty acid oxidation was estimated by the fitting of a biexponential function to the myocardial clearance of radioactivity (18).

### Ceramides

Ceramides from serum and adipose tissue were extracted using the butanol–methanol methods (19, 20). Adipose tissue ceramides were then further purified using straight-phase HPLC, coupled to a fraction collector (21). Ceramides from both serum and adipose tissue were detected and quantified using ultraperformance liquid chromatography/tandem mass spectrometry, as previously described (22).

### Dietary intake, physical activity, and compliance

Dietary intake was assessed by 4-day weighed food records (at baseline and week 8) and processed with DietistNET dietary assessment software. During this 4-day periods, subjects wore accelerometers (Philips Respironics Actical) on their right ankle to assess 24-hour physical activity. Fatty acid composition was measured in the intervention oils used in the isocaloric food items, as well as in plasma cholesterol esters (CEs), phospholipids (PLs), and adipose tissue triglycerides by gas chromatography, as previously described (23). Subcutaneous adipose tissue biopsies were taken as previously described (3).

SCD activity was estimated as the 16:1n-7/16:0 ratio. Delta-5 desaturase activity was estimated as the 20:4n-6/20:3n-6 ratio and delta-6 desaturase as the 18:3n-6/18:2n-6 ratio.

### Clinical and laboratory analyses

Participants completed an oral glucose tolerance test by consuming 75 g glucose dissolved in 200 mL water (blood sampling at 0, 30, 60, and 120 minutes). Glucose, total cholesterol, high-density lipoprotein (HDL)-cholesterol, low-density lipoprotein (LDL)-cholesterol (directly measured), triglycerides, C-reactive protein, apolipoprotein (apo)B, and apoA1 were analyzed in plasma and insulin in serum by standard laboratory methods at Uppsala University Hospital.

Vascular cell adhesion molecule-1, intercellular adhesion molecule-1, endostatin, and TNF receptor 1 and 2 (TNF-R1 and TNF-R2) were analyzed in plasma by ELISA (R&D Systems). d-3-Hydroxybutyrate was analyzed using a kinetic enzymatic method using the Ranbut reagent (RB1008; Randox Laboratories).

### Cholesterol metabolism and hepatokines

Serum fibroblast growth factor (FGF)21, proprotein convertase subtilisin/kexin type 9 (PCSK9), and FGF19 levels were determined by ELISA (Catalog nos. DF2100, DPC900, and DF1900, respectively; R&D Systems), according to the manufacturer’s instructions. Serum NEFA levels were determined using an enzymatic colorimetric assay [HR Series NEFA-HR(2); Wako Diagnostics]. Levels of serum BAs and 7a-hydroxy-4-cholesten-3-one (C4), a BA synthesis marker, were determined by liquid chromatography–tandem mass-spectrometry using deuterium-labeled standards for C4 and BA (24). Unesterified lathosterol, a serum marker of total cholesterol synthesis, was determined by gas chromatography–mass spectrometry (25). C4 and lathosterol levels were corrected for total serum and total cholesterol (C4c and lathosterol/c), respectively (26, 27).

### Statistical analysis

Differences in mean values between groups postintervention were analyzed with ANCOVA, adjusted for baseline values. Differences in mean values between subgroups (*e.g.*, for PET-MR) were analyzed with Mann-Whitney test. Data are given as means (SD) or medians [interquartile range (IQR)]. Correlations between variables are given as Pearson’s r or Spearman’s rho. A *P* value <0.05 was considered statistically significant. JMP version 13.1.0 was used; heatmaps were created using R. Primary outcome measure for the trial was change in lean tissue mass (reported in a separate manuscript); secondary outcome measures included changes in liver and pancreas fat, VAT, total body fat, palmitate uptake, and blood lipids. Sample size was determined using Lehr formula, based on our previous trial, using similar intervention and outcome measures (3). For lean tissue, n = 22 subjects per group was needed to detect a 0.55-L difference between groups, with *α* = 0.05 and *β* = 0.20. For liver fat, n = 23 subjects per group was needed to detect a 0.52% units difference between groups with *α* = 0.05. For the exploratory assessment of hepatic palmitate uptake, we had no previous data to use for sample-size determination. With the current results in hand, n = 13 subjects per group would have been needed to detect a 20% difference in net uptake rate between groups with *α* = 0.05.

### Ethics

This study was conducted in accordance with the Declaration of Helsinki. All subjects provided written, informed consent before inclusion, and the study was approved by the Regional Ethical Review Board in Uppsala (Dnr 2014/186). All authors reviewed and approved the final manuscript.

## Results

Sixty subjects (n = 30 in each group) completed the 8-week hypercaloric period; baseline characteristics are shown in Table 1. Age (42.3 ± 9.5 vs 41.6 ± 7.3, *P* = 0.75), BMI (28.3 ± 3.5 vs 27.7 ± 3.9, *P* = 0.52), and sex distribution (12:18 vs 11:19 women/men) were similar between SFA and PUFA groups, respectively. By design, surplus energy intake and macronutrient distribution were also similar between groups, resulting in similar (*P* = 0.50) body weight gain [2.31 ± 1.38 kg (2.6 ± 1.5%) vs 2.01 ± 1.90 kg (2.5 ± 2.3%) for SFA and PUFA, respectively]. Baseline, 24-hour physical activity was similar (*P* = 0.32) between groups and did not change during intervention (data not shown).

**Table 1.** Baseline Characteristics

	SFAFA Group (n = 30)	PUFA Group (n = 30)	PET-MR Subgroup Within SFA Group (n = 5)	PET-MR Subgroup Within PUFA Group (n = 5)
Age, y	42 ± 10	42 ± 7	43 ± 12	39 ± 7
Men/women	18/12	19/11	5/0	5/0
BMI	28.3 ± 3.5	27.7 ± 3.6	26.2 ± 1.4	26.5 ± 1.5
Systolic blood pressure, mm Hg	121 ± 14	117 ± 12	120 ± 8	121 ± 12
Diastolic blood pressure, mm Hg	78 ± 10	73 ± 9	76 ± 7	73 ± 8
Glucose, mM	5.6 ± 0.4	5.7 ± 0.5	5.9 ± 0.4	5.8 ± 0.4
Triglycerides, mM	1.0 ± 0.3	1.1 ± 0.4	0.8 ± 0.3	1.0 ± 0.3
Total cholesterol, mM	4.5 ± 0.9	4.7 ± 0.8	4.6 ± 1.9	4.5 ± 0.6
LDL-cholesterol, mM	2.75 ± 0.80	3.03 ± 0.69	2.67 ± 1.57	2.96 ± 0.60
HDL-cholesterol, mM	1.3 ± 0.3	1.2 ± 0.3	1.3 ± 0.3	1.1 ± 0.1
ALT, µkat/L	0.45 ± 0.27	0.48 ± 0.32	0.58 ± 0.42	0.66 ± 0.51
HOMA-IR	2.3 ± 1.1	2.2 ± 1.2	1.9 ± 0.2	2.3 ± 1.0
Liver fat, %	1.46 (0.96–3.89)	2.02 (1.36–4.56)	1.29 (1.01–1.48)	1.54 (1.37–9.70)
Visceral fat, L	3.60 ± 2.23	3.34 ± 1.82	3.37 ± 1.44	3.70 ± 1.11
Total body fat, %	32.2 ± 8.6	30.4 ± 10.2	26.7 ± 4.4	25.8 ± 5.5
Pancreas fat, %	4.81 ± 4.77	5.35 ± 6.44	6.07 ± 2.96	5.90 ± 4.34
NEFA, mM	0.31 ± 0.15	0.29 ± 0.14	0.30 ± 0.16	0.22 ± 0.11

Data are means (SD) or median (IQR).

Abbreviations: ALT, alanine aminotransferase; HOMA-IR, homeostatic model assessment of insulin resistance.

### Compliance to diets

Changes in fatty acid composition in both plasma and adipose tissue reflected the assigned interventions, indicating high compliance (28). Linoleate, the major fatty acid in the PUFA muffins, increased in plasma CE, PL, and subcutaneous adipose tissue in the PUFA group (*P* < 0.0001 for comparison between groups), whereas palmitate, the major fatty acid in the SFA muffins, increased in both plasma PL and adipose tissue in the SFA group during the intervention (*P* < 0.0001 for difference between groups). In addition, based on checklists/self-report, subjects in the SFA group consumed 96.8%, and subjects in the PUFA group consumed 97.1% of the provided muffins, and the 4-day weighed dietary records indicated no changes other than those desired/expected in fat type. Finally, cholesterol levels changed in accordance to what would be expected based on increased intakes of SFA and PUFA, respectively (*vide infra*). Thus, both key dietary biomarkers, as well as self-reported data, suggested excellent compliance to both diets.

### Lipid profile and cholesterol metabolism

**Figure 1.**
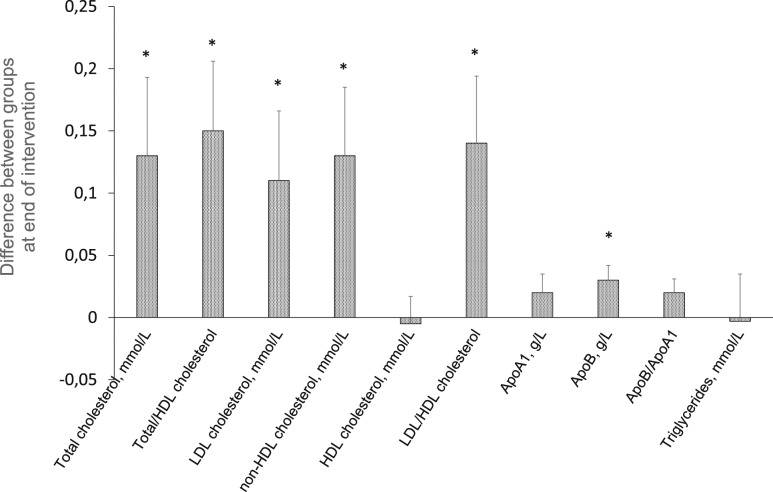
Difference in blood lipids between groups at end of intervention, adjusted for baseline values. Differences represent “SFA vs PUFA”; *i.e.*, a positive change means the variable was higher on the SFA-rich diet. Bars are SE. Analyzed with ANCOVA. *P < 0.05.

SFA overfeeding deteriorated the blood lipid profile (*e.g.*, increasing LDL-cholesterol and apoB), whereas PUFA overfeeding reduced atherogenic blood lipids, despite weight gain (Fig. 1) (28). Compared with SFA, PUFA caused a reduction (*P* = 0.004) in circulating lathosterol, a marker of cholesterol synthesis; this was, however, attenuated when corrected for total cholesterol, indicating no change in cholesterol synthesis, in line with unchanged PCSK9. Neither fasting serum total BAs nor their composition or other markers of cholesterol and BA synthesis (*e.g.*, FGF19 and C4) differed between diets (28). Serum total BAs increased postprandially in both groups, but areas under the curve did not differ between groups.

### Liver and pancreas fat

**Figure 2.**
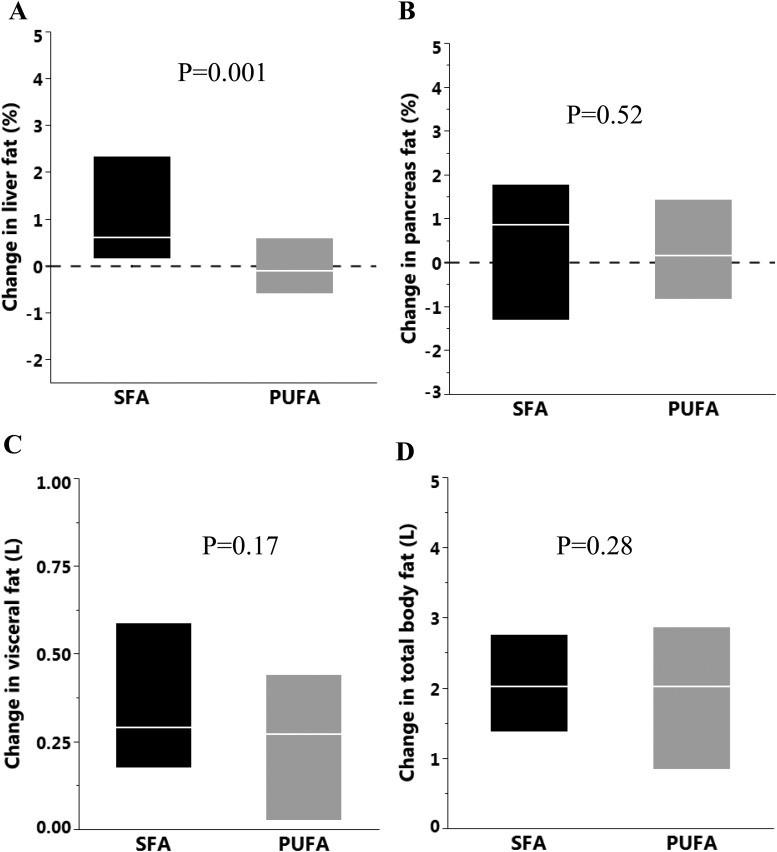
Change in (A) liver fat (percentage points), (B) pancreas fat (percentage points), (C) visceral fat, and (D) total body fat in the SFA (n = 30) and PUFA (n = 30) groups. The boxes represent the IQR and the lines within the median. Analyzed with ANCOVA, adjusted for baseline value.

Liver fat increased by 53% (1.54 ± 2.0% points; 30 ± 40 mL) in the SFA group, whereas in the PUFA group, there was a 2% decrease (−0.09 ± 1.55% points; −1 ± 30 mL; *P* = 0.001 for between-group difference), despite similar body-weight gain (Fig. 2A). The different liver fat accumulation was reflected by plasma alanine aminotransferase (ALT) levels, which increased by 18% (Δ0.08 ± 0.18 µkat/L; from 0.45 ± 0.27 µkat/L to 0.53 ± 0.29 µkat/L) in the SFA group and remained unchanged (Δ−0.01 ± 0.14 µkat/L; from 0.48 ± 0.32 µkat/L to 0.47 ± 0.27 µkat/L) in the PUFA group (*P* = 0.035 for between-group difference). The change in liver fat was inversely correlated with change in circulating linoleate in both plasma (r = −0.48, *P* = 0.0001 for CE and r = −0.38, *P* = 0.003 for PL) and adipose tissue (r = −0.32, *P* = 0.04) but directly associated with change in the circulating palmitate (r = 0.30, *P* = 0.02 and r = 0.52, *P* = 0.0001 for CE and PL, respectively) and SCD activity index in CE (r = 0.49, *P* = 0.0001). The changes in liver fat did not translate to significant changes in glucose tolerance or insulin sensitivity between groups (28).

Pancreas fat accumulation was similar (*P* = 0.52) in SFA (0.49 ± 2.29%) and PUFA (0.46 ± 1.69%) groups (Fig. 2B).

### Visceral and total body fat

The accumulation of VAT was similar (*P* = 0.17) in SFA (0.37 ± 0.29 L) and PUFA (0.26 ± 0.30 L) groups, as was the accumulation of total body fat (2.22 ± 1.57 L vs 1.77 ± 1.63 L, respectively, *P* = 0.28; Fig. 2C and 2D).

### Hepatic palmitate uptake by PET-MRI

In the exploratory subgroup study using PET-MRI, the change in hepatic palmitate uptake did not differ between groups, neither when expressed as SUVs at 30 or 60 minutes nor when assessing net influx rate K_i_ during different time intervals (28). Change in liver fat in this subgroup reflected that seen in the full sample. (Liver fat quantification failed at one occasion; hence, this comparison is for n = 4 vs n = 5.) In both groups combined (n = 10), none of the measurements of palmitate uptake changed during the intervention (data not shown). The change in hepatic palmitate uptake (net uptake rate K_i_ 5 to 60 minutes) tended to be inversely associated with change in liver fat, but there was no association between hepatic palmitate net uptake rate and liver fat at baseline (r = 0.18, *P* = 0.61; data not shown).

### Ceramides and sphingomyelins

**Figure 3.**
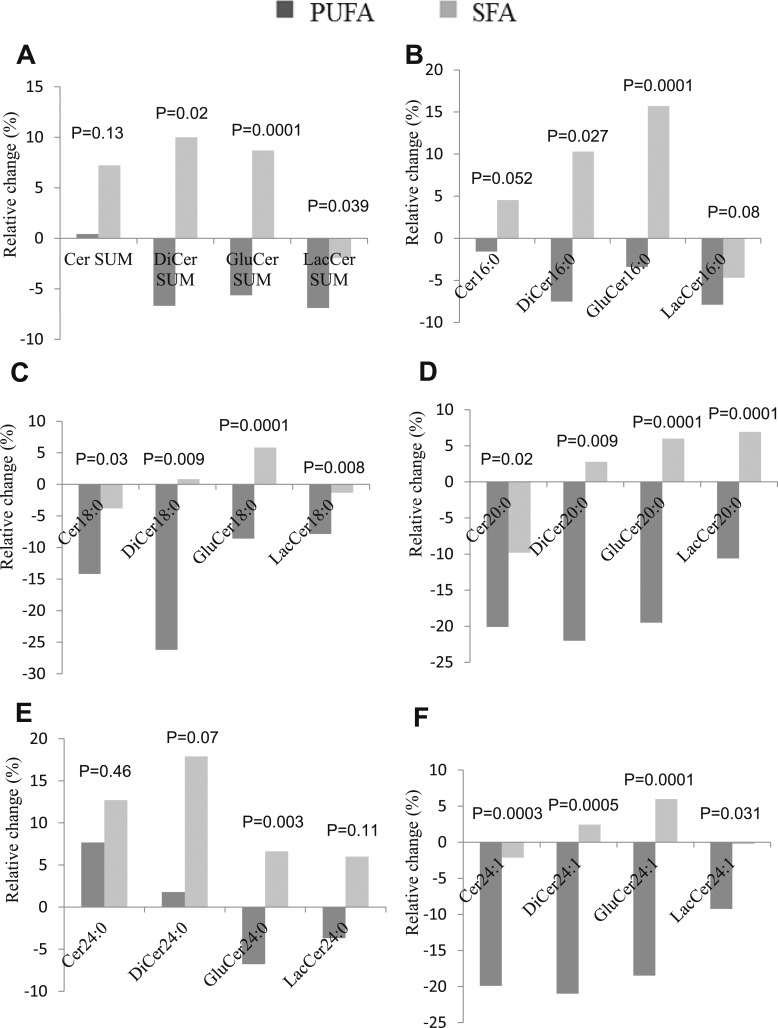
Relative change from baseline (%) in serum. (A) Total ceramide species, (B) C16-containing ceramide species, (C) C18-containing ceramide species, (D) C20-containing ceramide species, (E) C24-containing ceramide species, and (F) C24:1-containing ceramide species in SFA (n = 30) and PUFA (n = 30) groups. Analyzed with ANCOVA, adjusted for baseline value.

SFA- and PUFA-enriched diets had markedly different effects on serum ceramides (Cer), dihydroceramides (DiCer), glucosylceramides (GluCer), and lactosylceramides (LacCer). Overall, SFA increased, whereas PUFA decreased ceramide levels—changes that were already evident at 4 weeks (Fig. 3). Change in liver fat was directly associated with change in species from all ceramide groups (28). Interestingly, the differential effects on liver fat by SFA- and PUFA-rich diets were diminished (*P* = 0.08) when adjusted for changes in C16-containing ceramides (Cer16:0, DiCer16:0, and GluCer16:0; data not shown). Changes in circulating 16:1n-7 and the SCD index were positively correlated with changes in multiple ceramide species, whereas changes in circulating linoleate showed consistent, inverse associations (28).

Although changes in adipose tissue ceramides were also generally different between SFA and PUFA groups, changes were less pronounced and with few exceptions, not statistically different between groups.

SFA and PUFA overfeeding had differential effects, also on circulating sphingomyelin species: 16:0, 16:1, 18:0, and 18:1 species increased significantly more by SFA (changes evident already at 4 weeks), and changes in 16:0 and 16:1 species were directly associated with changes in both liver and VAT fat levels (r ∼ 0.3, *P* < 0.03; data not shown).

### Palmitate uptake in pancreas, heart, and skeletal muscle

Changes in palmitate uptake in pancreas, skeletal muscle, or heart did not differ between groups (28). In both groups combined, none of the measurements changed as a result of the intervention (data not shown). Myocardial palmitate uptake or oxidation did not differ between groups (28), but uptake increased by 40% during the intervention (*P* = 0.037) in both groups combined (data not shown).

**Figure 4.**
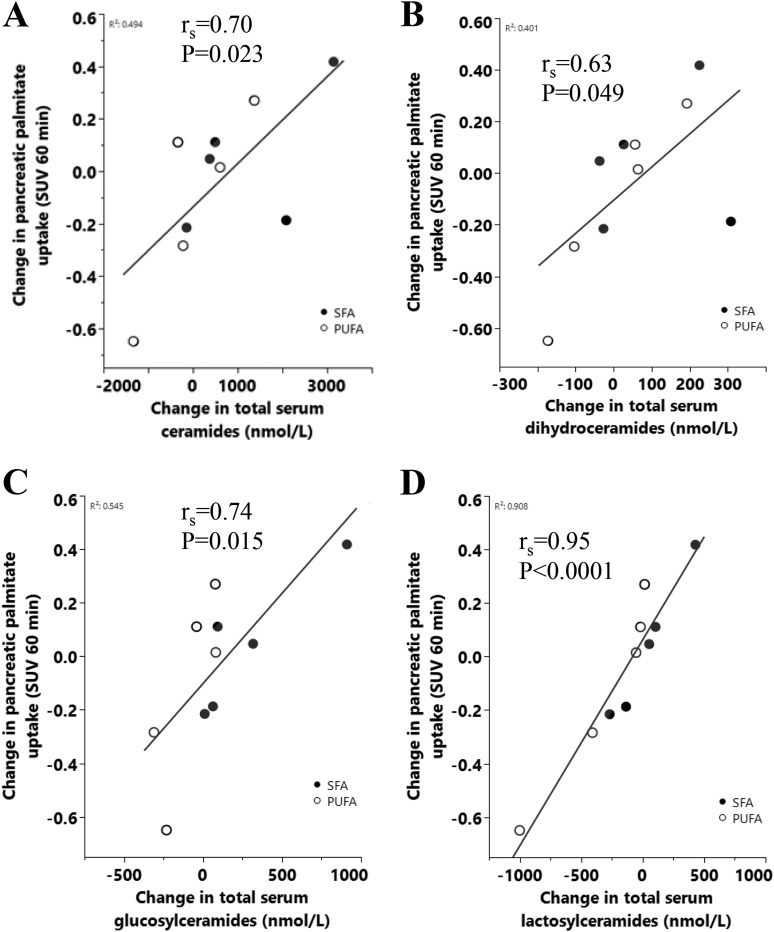
Spearman correlations between change in pancreatic palmitate uptake (SUV 60 min) and change in (A) total serum Cer, (B) total serum DiCer, (C) total serum GluCer, and (D) total serum LacCer in 10 men.

Changes in serum Cer, DiCer, GluCer, and LacCer were strongly, positively associated (r = 0.63 to 0.95), with changes in pancreatic (but not hepatic) palmitate uptake assessed as SUV at 30 and 60 minutes (Fig. 4A–4D). Similar but attenuated associations were observed when palmitate uptake was assessed as net uptake rate K_i_. Furthermore, similar results were observed for the individual C16 and C18 species (*P* = 0.002 to 0.03 and *P* = 0.001 to 0.07, respectively; data not shown).

### Inflammation, oxidative stress, and endothelial function

We did not observe any differences in markers of inflammation (C-reactive protein, TNF-R1, TNF-R2), endothelial function (vascular cell adhesion molecule-1, intercellular adhesion molecule-1, endostatin), or free radical-induced lipid peroxidation (isoprostane 8-iso-prostaglandin F2*α*) as a result of PUFA compared with SFA overfeeding (28).

### Calorie restriction and diet-induced weight loss

Fifty-one subjects (n = 26/25 from SFA and PUFA groups, respectively) completed the 4-week hypocaloric period (∼800 kcal/d), which caused similar (*P* = 0.92) weight loss (−4.55 ± 2.39 kg and −4.48 ± 2.63 kg, respectively). Furthermore, both groups decreased similarly (*P* = 0.82) in waist circumference (−4.62 ± 5.68 vs −4.26 ± 5.29 cm, respectively) and total body fat (kilograms, ADP; −3.70 ± 2.61 vs −3.21 ± 2.57 kg, respectively; *P* = 0.94). All differences observed during weight gain between groups in blood lipids were abolished by weight loss. Likewise, no differences between groups were observed for liver enzymes or ceramides after weight loss (data not shown). In both groups combined, changes in ceramides and sphingolipids showed diverse effects in response to weight loss. Total serum BAs (*P* = 0.012) and FGF19 (*P* = 0.042; a circulating inhibitor of BA synthesis) were higher in the SFA vs PUFA group after weight loss.

## Discussion

In a double-blind and randomized trial in overweight individuals, we show that overeating SFA from palm oil causes pronounced liver fat accumulation. Concomitantly, both circulating liver enzymes and ceramides increased, indicating hepatocellular injury. In contrast, despite similar weight gain, overeating PUFA from sunflower oil completely blocked liver fat accumulation and even improved the blood lipid profile. Importantly, adverse metabolic effects, induced by SFA or PUFA, normalized after weight loss.

The differential effects of SFA and PUFA on liver fat and blood lipid levels were even more distinct in the present overweight and middle-aged population compared with our previous study population consisting of young, lean subjects (3). Notably, the >50% relative increase in liver fat by SFA corresponded to a 30-mL actual increase in liver fat, whereas overfeeding PUFA did not alter liver fat. If anything, there was a slight decrease in liver fat by PUFA; these results are in line with our previous findings showing decreased liver fat by PUFA during weight-stable conditions in obese subjects (2). Accordingly, serum ALT increased after overfeeding SFA in contrast to PUFA. These effects accord with observational data showing inverse associations between PUFAs and liver fat in cross-sectional (23, 29) and longitudinal analyses (29), whereas SFAs show the opposite associations (29).

In retrospect, it was discovered that the subgroups used for exploratory analysis of hepatic palmitate uptake were underpowered. However, despite clear indications of differential liver fat accumulation in these subgroups, we observed no indications at all that hepatic palmitate uptake was differentially affected between diets. Dissociation between hepatic fatty acid uptake and liver fat content was recently observed by Immonen *et al.* (30), showing that elevated hepatic fatty acid uptake was maintained in individuals with obesity, 6 months after surgery-induced weight loss despite normalization of liver fat Taken together, this implies that factors other than palmitate uptake [*e.g.*, hepatic DNL and/or fatty acid oxidation, ketone body production, and secretion as very LDL (31)] may be more important for regulation of liver fat accumulation. Neither fasting nor postprandial d-3-hydroxybutyrate was differentially affected by diets, implying that hepatic fat oxidation may not be the primary mediator of differential effects on liver fat. It was recently suggested that adipose tissue lipolysis may partly explain the differential effects of SFA and unsaturated fat on overfeeding-induced liver fat accumulation (10); however, we observed no differences in fasting or postprandial NEFA between groups. The contributions from different fatty acid sources to the liver change from the fasted to the fed state; *e.g.*, adipose-derived NEFA decrease and chylomicron- and spillover-derived fatty acids increase their contribution in the fed state (32). Neither of these pathways was measured in the current study but would have been interesting to do, as they may potentially explain some of the difference in liver fat accumulation. Taken together, the mechanisms behind the differential effects on liver fat by SFA and PUFA remain elusive (33).

There were distinct effects on the lipoprotein profile between diets, more clearly than previously shown in normal-weight, young subjects (34). In contrast to SFA, PUFA reduced several blood lipids, despite the hypercaloric condition, demonstrating the strong impact of dietary fat quality on lipoprotein levels. Such effects may involve altered cholesterol synthesis, as circulating lathosterol, a marker for cholesterol synthesis (25, 26), was lower after PUFA vs SFA. Neither total BAs nor their composition was different between groups, suggesting that BAs are not a major mediator of the differential effects of SFA vs PUFA on liver fat content.

Ceramides could play a causal role in the pathogenesis of diet-induced NAFLD (14). In the current study, SFA and PUFA consistently showed opposite effects on circulating ceramides. Overall, SFA increased, whereas PUFA decreased ceramides, and changes in many of the individual species correlated directly with liver fat accumulation. In a recent overfeeding study, SFA caused more liver fat deposition than unsaturated fat, with a parallel increase in circulating ceramides (10). The liver in NAFLD associated with insulin resistance has previously been shown to be enriched with ceramides and SFA (35). Furthermore, changes in many ceramide species were positively associated with changes in circulating fatty acid 16:1n-7 and SCD, proposed markers of hepatic DNL, supporting a connection between hepatic DNL and ceramide synthesis (14, 36, 37). As we found fewer changes of ceramides in adipose tissue, the main effects of dietary palmitate probably mainly reflect hepatic ceramide production, as supported by the relationships with liver fat accumulation. In addition, ceramide species in plasma correlate with the respective species in human livers (38). An increased intake of palmitate is expected to play a key role in the ceramide synthesis rate generated by the *de novo* synthesis pathway, but mechanistic data are lacking in humans. In experimental models, a reduction in ceramides decreases both hepatic DNL and liver fat (14, 36, 37), whereas an increase in ceramide levels increases liver fat (13). In contrast, linoleic acid was inversely associated with changes in many ceramide species in the current study. Interestingly, the differential effects on liver fat accumulation by SFA and PUFA were abolished when adjusting for changes in C16 ceramides, supporting a crucial role for ceramides in SFA-induced steatosis also in humans. In addition to a stimulatory role of ceramides on hepatic DNL, experimental studies have demonstrated a stimulatory role on hepatic lipid uptake mediated via atypical protein kinase C activity on CD36 (14). Although statistically underpowered, our PET-MR scans did not indicate differential hepatic palmitate uptake between groups, suggesting that factors other than lipid uptake are more important for a potential steatosis-promoting effect of ceramides in humans.

We found strong associations between changes in all ceramide classes and change in pancreatic, but not hepatic, palmitate uptake. The significance of these correlations is unclear and needs to be confirmed.

In contrast to our previous study (3), accumulation of VAT and total body fat was not different between diets. Previous studies in humans with obesity have shown favorable effects of PUFA on abdominal fat (39, 40). Furthermore, the differential effects of SFA and PUFA on body fat content have been shown in animal models (41–43), although the mechanisms are yet unclear.

Despite robust differences in liver fat accumulation, no differences in measures of insulin resistance were observed between SFA and PUFA groups, although there was a moderate association between increased liver fat and markers of insulin resistance within the SFA group. A lack of differential effect between fat types on insulin sensitivity accords with our findings in lean individuals, and may be explained by the fact that the majority of subjects had a nonfatty liver (<5%) also after the intervention; *i.e.*, the absolute amount of liver fat accumulation may have been too small to impair insulin action also in the current metabolically healthy, overweight population, as supported by findings in obesity-matched subjects with and without NAFLD (44). Likewise, a small, randomized study (45) indicated that a mono-unsaturated fatty acid-induced reduction in liver fat did not improve insulin sensitivity, implying that changes in liver fat need to be larger and/or more long term to reduce insulin sensitivity. Although NAFLD may promote hepatic insulin resistance (1), there are also other examples of diet-induced moderate liver fat accumulation without concomitant impairment of insulin sensitivity (46).

SFA has been suggested to induce inflammation (47), based primarily on experimental studies. Although we found no circulating evidence that plasma markers of low-grade inflammation were differentially influenced between groups, it is possible that organ-specific effects may have occurred, as it was recently shown that saturated, but not unsaturated, fat upregulated inflammation-related genes in adipose tissue in humans (10). It should also be noted, that despite the high and hypercaloric intake of *n*-6 PUFA in the form of linoleate (a precursor to arachidonic acid), there were no signs of increased inflammation or lipid peroxidation, as judged by established plasma markers of inflammation and oxidative stress compared with SFA. This finding accords with results from our previous trials (2, 34) and thus, suggests no evidence for possible proinflammatory effects of dietary *n*-6 PUFA from sunflower oil.

A strength of this study is the double-blinded study design, which is rather unique for diet trials. In addition, measured changes in plasma and adipose tissue fatty acid composition suggested excellent adherence to both diets. Only one subject dropped out, thus minimizing attrition bias. Both dietary oils were of vegetable origin, excluding potential effects of differential cholesterol intake on liver fat (48). Finally, the study population was middle aged and overweight, demonstrating that the adverse effect of SFA and beneficial effects of PUFA on liver fat are relevant also in this common, high-risk population. The current findings are thus highly relevant for public health, considering the large proportion of adults that are susceptible to energy excess and the liver fat-promoting and cholesterol-raising effect of high SFA intake. The strong agreement between this study and our previous data (2, 3, 23) provides validity and reproducibility of these results. However, our study has some limitations. The used MRI methods relied on fixed-spectrum models and thus, did not allow full characterization of all lipid resonances of the liver spectra to detect changes in liver lipid saturation. Furthermore, the exploratory analyses on hepatic palmitate uptake were done only in small subgroups and should therefore be viewed as strictly hypothesis generating, and as the SUV measurements for palmitate uptake are affected by blood flow, it can therefore not be excluded that some results for SUV are because of altered blood flow. Finally, plasma NEFA (used for palmitate uptake calculations) was not collected at the time of the PET-MR scan but within ±1 day of the scan.

In summary, overfeeding SFA promotes liver fat accumulation in overweight humans, whereas overfeeding PUFA does not alter liver fat, despite similar weight gain. We also provide evidence that SFA and PUFA have opposite effects on circulating ceramides, suggesting a role for dietary fat type in modulating ceramide levels in humans. Notably, ceramides were associated with liver fat accumulation and proposed markers of hepatic DNL. Importantly, the differential cardiometabolic effects of SFA and PUFA during diet-induced weight gain were effectively abolished by subsequent caloric restriction. This study provides metabolic insights in the adverse role of SFA in diet-induced ectopic fat deposition and demonstrates the potential importance of replacing dietary SFA with PUFA (linoleate) for the prevention of NAFLD and hyperlipidemia.

## Abbreviations:

ADP: air displacement plethysmography
ALT: alanine aminotransferase
apo: apolipoprotein
BA: bile acid
BMI: body mass index
C4: 7a-hydroxy-4-cholesten-3-one
CE: cholesterol ester
Cer: ceramide
DiCer: dihydroceramide
DNL: *de novo* lipogenesis
FGF: fibroblast growth factor
GluCer: glucosylceramide
HDL: high-density lipoprotein
IQR: interquartile range
K_i_: inhibitory constant
LacCer: lactosylceramide
LDL: low-density lipoprotein
MR: magnetic resonance
NAFLD: nonalcoholic fatty liver disease
NEFA: nonesterified fatty acid
PCSK9: proprotein convertase subtilisin/kexin type 9
PET: positron emission tomography
PL: phospholipid
PUFA: polyunsaturated fatty acid
SCD: stearoyl-coenzyme A desaturase
SFA: saturated fatty acid
SUV: standardized uptake value
TNF-R: TNF receptor
VAT: visceral adipose tissue
